# Medical Lexicon

**Published:** 1849

**Authors:** 


### ARTICLE VII.

*Medical Lexicon of Modern Terminology.* Being a complete Vocabulary of Definitions, including all the Technical Terms employed by Writers and Teachers of Medical Science at the present day, and comprising several hundreds of words not found in any other Dictionary. Designed for the use of Students and Practitioners. Second Edition, greatly enlarged. By D. Meredith Reese, M. D., L. L. D., Resident Physician of Bellevue Hospital New York, Editor of Cooper's Surgical Dictionary, etc. pp. 233, 18mo. New York: S. S. and W. Wood. 1848. (From the Publishers, by Express.)

From the extent of title page used in setting forth the character of this work, one would be induced to expect to find it, a huge overgrown dictionary, filling volumes. But, instead of this, it is a neat little book, that you can buy for a small sum and carry in your pocket without inconvenience. We have had the pleasure of occasionally referring to it for a definition, and it has, in every case, proved satisfactory. The call for a second edition speaks well for its merits. E.

